# Cutaneous Tuberculosis Presenting as Mastitis in a Prepubertal Girl

**DOI:** 10.4269/ajtmh.18-0413

**Published:** 2018-12

**Authors:** Catalina Arango-Ferreira, Claudia M. Zapata-Muñoz, Eduardo Gotuzzo

**Affiliations:** 1 Division of Pediatric Infectious Diseases, Hospital San Vicente Fundación Medellín, Colombia;; 2 Department of Pediatrics, Medical School, University of Antioquia, Medellín, Colombia;; 3 Resident in Pediatrics, Medical School, University of Antioquia, Medelllín, Colombia;; 4 Instituto de Medicina Tropical Alexander von Humboldt, Universidad Peruana Cayetano Heredia, Lima, Perú

**Figure 1.**
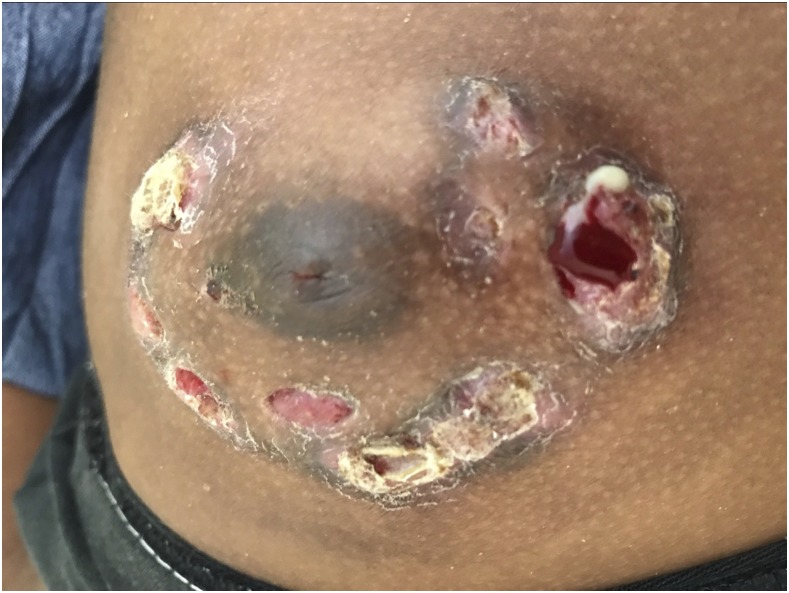
Left breast with inflammation, chronic ulcerations, abscesses with purulent discharge, and scars. This figure appears in color at www.ajtmh.org.

**Figure 2.**
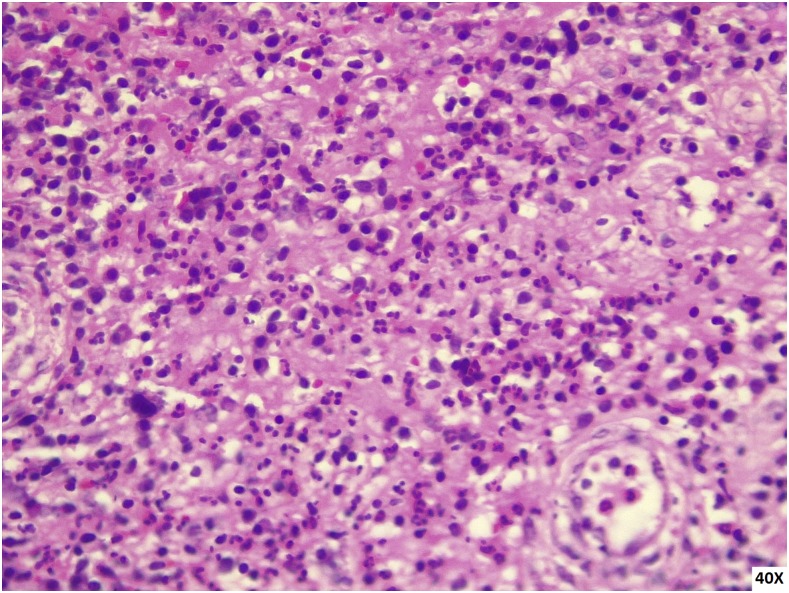
Histopathology photograph showing granuloma, chronic inflammation. This figure appears in color at www.ajtmh.org.

**Figure 3.**
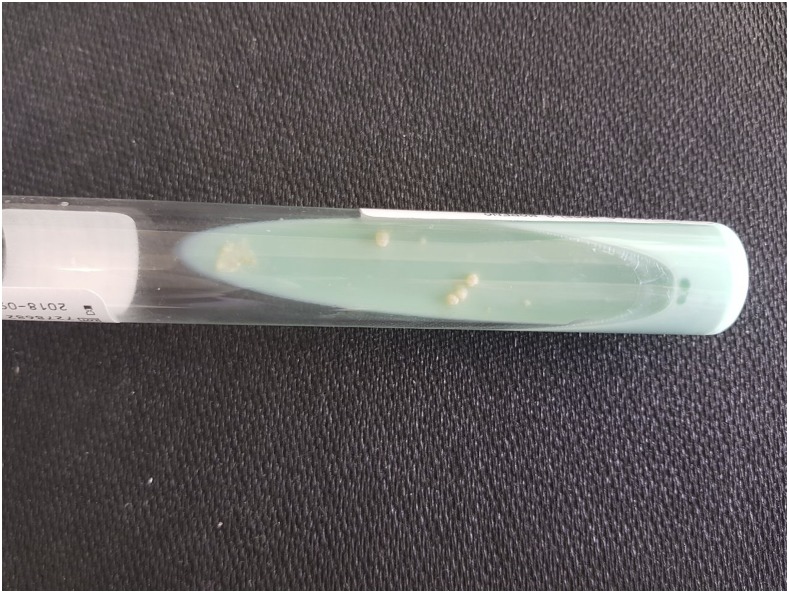
Positive mycobacterial culture on solid media. This figure appears in color at www.ajtmh.org.

A 9-year-old girl, from a rural area of Chocó in Colombia, had 10 months of multiple lesions involving the left breast. She denied previous trauma, but often had swum in rural fresh water ponds. The lesions were reported to have started as a single hyperpigmented brown non-painful plaque that became a papule, and then a pustule. Fistulization followed, and then purulent drainage. Similar lesions appeared around the areola with scarring and chronic ulceration. The lesions recurred and were painful. Empiric oral and intravenous antibiotics were prescribed without improvement. Physical examination showed the breast to be swollen and tender. Ten lesions around the areola were seen, a combination of scars, ulcers, abscesses, and cutaneous fistulas (Figure 1). The right breast was unaffected. Chest x-ray and complete blood counts were normal; a fourth-generation test for the human immunodeficiency virus-1 was negative. Three pus samples obtained by digital compression were negative by Ziehl-Neelsen, Kinyoun, and Gomori methenamine–silver stains (Figure 2). A skin biopsy showed giant cell granulomas without caseation and acute inflammation. By the time the patient was reexamined 4 weeks later, all three samples yielded positive mycobacterial cultures on solid media (Figure 3). A rapid lateral flow assay (SD BIOLINE TB Ag MPT64 Rapid TEST) was consistent with *Mycobacterium tuberculosis* complex, further confirmed by polymerase chain reaction (GenoType Direct mycobacterial assay, Anyplex micobacterium tuberculosis/non tuberculous micobacteria assay). Primary cutaneous tuberculosis (TB) was confirmed. After mycobacteriological sensitivities were confirmed, the patient was started on standard four-drug therapy with isoniazid, rifampin, pyrazinamide, and ethambutol for 2 months, and isoniazid and rifampin after that to complete 6 months of therapy. Three household neighbors were diagnosed with pulmonary TB after a home investigation.

Tuberculosis affecting children was approximately 7% of all newly reported cases in 2016 worldwide.^1^ Cutaneous TB is an uncommon form of extrapulmonary TB, representing 1–2% such infections. Skin manifestations can occur through three different mechanisms, which are related to clinical presentation: 1) exogenous inoculation (verrucosa cutis); 2) dissemination of an endogenous infection such as scrofuloderma, osteomyelitis, or TB orificialis by autoinoculation; or 3) hematogenous spread from a distant infection site as miliary TB.^2–4^ Differential diagnoses include environmental mycobacteria, leprosy, cutaneous leishmaniasis, *Nocardia* spp., actinomycosis, deep fungal infections, and idiopathic granulomatous mastitis.^5,6^ Diagnosis is confirmed through combination of stains, cultures, and molecular tests, which determines treatment.
